# Supplementary material for: Aspiletrein A Induces Apoptosis Cell Death via Increasing Reactive Oxygen Species Generation and AMPK Activation in Non-Small-Cell Lung Cancer Cells
Source: Int J Mol Sci. 2022 Aug 17;23(16):9258. doi: 10.3390/ijms23169258 (PMC9409406; doi:10.3390/ijms23169258)
Supplement: Supplementary file 1 [file ijms-23-09258-s001.zip › ijms-1841948-supplementary.pdf]

Supplementary information for

Aspiletrein A induces apoptosis cell death via increasing reactive oxygen species generation and AMPK activation in non-small cell lung cancer cells

Wasita Witayateeraporn[1], Hien Minh Nguyen[2], Duc Viet Ho[3], Hoai Thi Nguy[3], Pithi Chanvorachote[1], Chanida Vinayanuwattikun[4], Varisa Pongrakhananon[1,5*]

[1] Department of Pharmacology and Physiology, Faculty of Pharmaceutical Sciences, Chulalongkorn University, Bangkok, 10330 Thailand

[2] Faculty of Pharmacy, Ton Duc Thang University, Ho Chi Minh City, 700000 Vietnam

[3] Faculty of Pharmacy, Hue University of Medicine and Pharmacy, Hue City, 49000 Vietnam

[4] Division of Medical Oncology, Department of Medicine, Faculty of Medicine, Chulalongkorn University, Bangkok, 10330 Thailand

[5] Preclinical Toxicity and Efficacy Assessment of Medicines and Chemicals Research Unit, Chulalongkorn University, Bangkok, 10330 Thailand

***Corresponding author**

Varisa Pongrakhananon, Department of Pharmacology and Physiology,

Faculty of Pharmaceutical Sciences, Chulalongkorn University, 254 Phayathai, Wangmai, Pathumwan, Bangkok, Thailand 10330

Tel: +662-218-8325; Fax: +662-218-8340

Email: Varisa.p@pharm.chula.ac.th

Supplementary materials and methods

Quantitative real time polymerase chain reaction (qRT-PCR)

A number of 3×10^5 cells/well were seeded onto 6-well plates for 24 h, and further treated with 25 and 50 µM of AA for 24 h. The mRNA was extracted by GENEzol reagent (Geneaid Biotech, New Taipei, Taiwan), and reverse transcribed by SuperScript™ III Reverse Transcriptase (Invitrogen, Carlsbad, CA, USA). A 20 µl of reaction was prepared by mixture of cDNA template, 2x SensiFAST™SYBR® No-ROX Kit, and primers. The mRNA expression of Bcl-2 was analyzed by using StepOnePlus Real-Time PCR system (Applied Biosystems, Loughborough, UK). The primers used were applied as following: *Bcl-2* forward primer: 5'-GCAGTGTGGTCTCCGAATGTC-3'; *Bcl-2* reverse primer: 5'-CATTGCCTCTCCTCACGTTCC-3'; *GAPDH* forward primer: 5'-ACATCGCTCAGACACCATG-3'; *GAPDH* reverse primer: 5'-TGTAGTTGAGGTCAATGAAGGG-3'. The expression levels were evaluated using $2^{-\Delta\Delta Ct}$ method.

Cell culture and selectivity index (SI) assessment

Human bronchial epithelial (BEAS-2B) cells were obtained from the American Type Culture Collection (ATCC; Manassas, VA, USA). Cells were cultured in DMEM, supplemented with 10% fetal bovine serum albumin, 2 mM L-glutamine, and 100 U/mL penicillin-streptomycin. Cell were maintained in 5% CO_2 at 37 °C. For selectivity index (SI) assessment, BEAS-2B cells were treated with various concentrations of AA for 24 h, and cell viability was examined by MTT assay. SI was calculated from IC_{50} of BEAS-2B cells to IC_{50} of cancer cells. The greater SI value indicates the higher selectivity to cancer [60].

Supplementary Figure S1

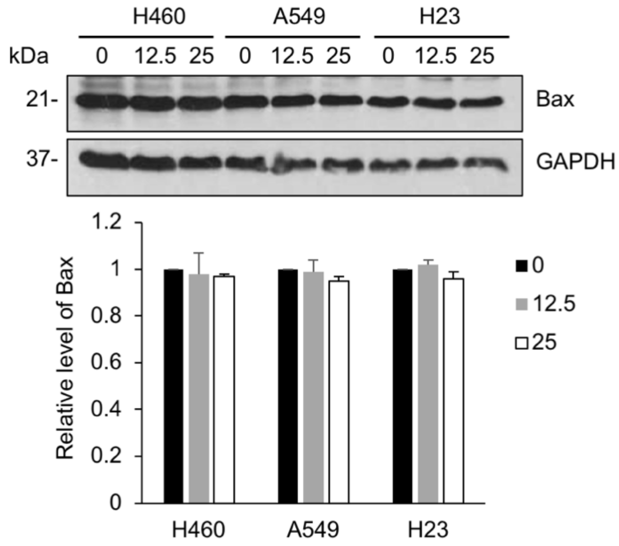

Figure S1. Effect of AA on Bax expression. The level of Bax was analyzed by western blotting. Blots were re-probed with anti-GAPDH to confirm equal loading. Protein level was quantified and presented as relative value to the control. Data are mean ± SEM (n = 4). $*p < 0.05$ vs control group.

Supplementary Figure S2

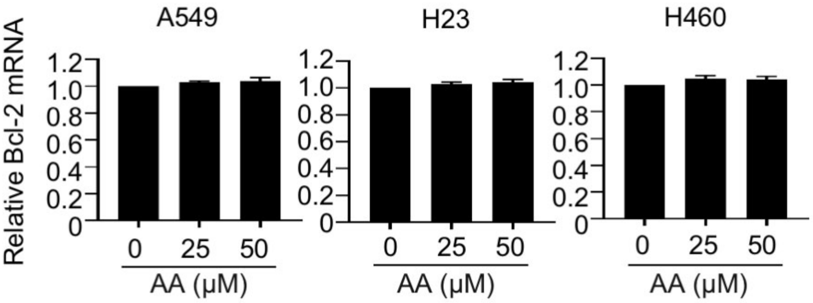

Figure S2. The effect of AA on Bcl-2 mRNA expression. Cells were treated with AA (0-50 μM) for 24 h. The mRNA was extracted and converted to cDNA. The mRNA level was analyzed by qRT-PCR using specific primer to Bcl-2 and GAPDH as loading control. Data are mean ± SEM from three independent experiments. * $p < 0.05$ vs control group.

Supplementary Table S1 Cytotoxicity of AA against lung cancer and normal lung epithelial cell lines.

Lung cancer cell lines	IC_{50} (µM ± SD)	Bronchial epithelial cell line	IC_{50} (µM ± SD)	SI
A549	9.60 ± 2.57	BEAS-2B	25.11 ± 4.73	2.6
H23	11.43 ± 3.07			2.19
H460	15.44 ± 3.29			1.62
